# Controversies in Midday Water Potential Regulation and Stomatal Behavior Might Result From the Environment, Genotype, and/or Rootstock: Evidence From Carménère and Syrah Grapevine Varieties

**DOI:** 10.3389/fpls.2019.01522

**Published:** 2019-12-02

**Authors:** Luis Villalobos-González, Mariana Muñoz-Araya, Nicolas Franck, Claudio Pastenes

**Affiliations:** ^1^Facultad de Ciencias Agronómicas, Universidad de Chile, Santiago, Chile; ^2^Programa de Doctorado en Ciencias Silvoagropecuarias y Veterinarias, Campus Sur Universidad de Chile, La Pintana, Chile

**Keywords:** water relations, *Vitis vinifera* L., drought, isohydricity, leaf traits

## Abstract

Controversies exist regarding the iso/anisohydric continuum for classifying plant water-use strategies. Isohydricity has been argued to result from plant–environment interaction rather than it being an intrinsic property of the plant itself. Discrepancies remain regarding the degree of isohydricity (σ) of plants and their threshold for physiological responses and resistance to drought. Thus, the aim of this study was to evaluate the isohydricity of the grapevine varieties Syrah and Carménère under a non-lethal water deficit progression from veraison from two different locations, the Cachapoal Valley (CV) and Maipo Valley (MV), in central Chile and with different rootstock only in Syrah. For this purpose, the midday stem water potential (Ψ_mds_) regulation and stomatal responses to drought, leaf traits related to pressure–volume curves, stomatal sensitivity to ABA, cavitation threshold, and photosynthetic responses were assessed. A higher atmospheric water demand was observed in the CV compared to the MV, with lower Ψ_mds_ values in the former for both varieties. Also, the σ values in Carménère were 1.11 ± 0.14 MPa MPa^-1^ and 0.68 ± 0.18 MPa MPa^-1^ in the CV and MV, respectively, and in Syrah they were 1.10 ± 0.07 MPa MPa^-1^ in the CV and 0.60 ± 0.10 MPa MPa^-1^ in the MV. Even though similar variations in σ between locations in both varieties were evident, Carménère plants showed a conserved stomatal response to Ψ_mds_ in both study sites, while those of Syrah resulted in a higher stomatal sensitivity to Ψ_mds_ in the site of lower σ. Besides the differences in seasonal weather conditions, it is likely that the different rootstock and clonal variability of each season in Syrah were able to induce coordinated changes in σ, Ψ_gs12_, and osmotic potential at full turgor (π_0_). On the other hand, irrespective of the σ, and given the similarity between the π_0_ and Ψ_gs12_ in leaves before drought, it seems that π_0_ could be a convenient tool for assessing the Ψ_mds_ threshold values posing a risk to the plants in order to aid the irrigation decision making in grapevines under controlled water deficit. Finally, water deficits in vineyards might irreversibly compromise the photosynthetic capacity of leaves.

## Introduction

The isohydric and anisohydric continuum was introduced initially as the divergent regulation of the midday leaf water potential (Ψ_md_) in plants (Berger-Landefeldt, 1936; Tardieu and Simonneau, 1998). Since it is generally recognized that a relationship exist in plants between the stomatal conductance and the leaf water status, isohydric plants have long been conceived as strictly conservative in their water balance, by reducing transpiration and water losses through reductions in stomatal conductance (g_s_), maintaining a nearly constant leaf water potential at midday (Ψ_md_), regardless of the soil water content (Steudle, 2000; Bucci et al., 2004). On the contrary, anisohydric plants have been extensively defined as less responsive at the stomatal level upon reductions in the soil water content, progressively reducing their Ψ_md_, but they are capable of maintaining transpiration and photosynthesis (Tardieu and Simonneau, 1998; Sade et al., 2012).

Therefore, to quantify the plant species position in the isohydric–anisohydric continuum, it has been proposed that the degree of isohydricity (σ) assessing the Ψ_md_ response as the soil water content is reduced (Martínez-Vilalta et al., 2014), among others (for a review see: Hochberg et al., 2018 and Feng et al., 2019). Also, the pre-dawn leaf water potential (Ψ_pd_) has been adopted as a *proxi* for soil water potential since transpiration is strongly reduced during the night and the hydraulic distribution removes gradients in Ψ among leaves and roots, reaching an equilibrium with the soil (Bauerle et al., 2008).

The degree of isohydricity is defined as the slope (σ) of the function describing the Ψ_pd_ to Ψ_md_ relationship in the abovementioned framework (Martínez-Vilalta et al., 2014) and it is assumed to be near zero for highly isohydric species and ≥ 1 for most anisohydric species. Also, it has been argued that plants with a high stomatal sensitivity, i.e. where stomatal conductance has been reduced at a relatively high water potential, could still show anisohydric behavior provided that its hydraulic transport system is more sensitive to declining water availability than stomata (Martínez-Vilalta et al., 2014). However, the evidence indicates that stomatal behavior in response to decreasing water potential is highly correlated with the beginning of xylem cavitation (Brodribb et al., 2003; Li et al., 2015; Bourne et al., 2017; Li et al., 2018) and leaf embolism in grapevines (Hochberg et al., 2017).

Recently, two observations have arisen: on one hand, a single plant might change from a higher to lower σ at a given water stress threshold (Poni et al., 2007; Meinzer et al., 2016; Charrier et al., 2018) and, on the other hand, the degree of isohydricity is not necessarily related to their stomatal sensitivity to drought across sites (Martínez-Vilalta and Garcia-Forner, 2016), highlighting the importance of the environment and, in both cases, challenging the diagnosis of isohydricity (Hochberg et al., 2018; Feng et al., 2019). Within a site, however, where plants experience similar VPD and edaphic conditions, it has been found that the isohydricity is a strong predictor of the stomatal response to drought between species (Novick et al., 2019).

As for drought tolerance, some relevant hydraulic traits have been defined based on the Ψ thresholds that trigger some physiological processes, like stomatal closure, the onset of embolism, or the water potential at the turgor loss point (Ψ_TLP_). Also, it has been proposed that some degree of association between these traits exists, and a better performance is expected upon drought in plants with low values for the abovementioned Ψ thresholds (Bartlett et al., 2012). Indeed, these traits have been consistently used for assessing the plant species’ susceptibility to prolonged drought in wild ecosystems (Skelton et al., 2015; Anderegg et al., 2016), but there is still no clear relationship between this and the paradigmatic isohydric–anisohydric continuum (Hochberg et al., 2018; Feng et al., 2019), even though some suggestions have been made (Tombesi et al., 2014; Meinzer et al., 2016; Meinzer et al., 2017).

On the other hand, more recently it has been reported that the abscisic acid (ABA), the concentration of which in the various plant parts increases upon water stress and plays a relevant role in the stomatal control (Sirichandra et al., 2009; Rogiers et al., 2012; Chater et al., 2014; McAdam and Brodribb, 2014), is under differential stomatal sensitivity between iso- and anisohydric varieties (Tardieu et al., 2015). It is in grapevines where ABA has also been indicated as a late response in stomatal control, following hydraulic signals (Tombesi et al., 2015; Rodriguez-Dominguez et al., 2016). Interestingly, it has been suggested that the differential sensitivity to ABA in grapevine varieties with contrasting degrees of isohydricity lies in the differential effect of the hormone on the leaf hydraulic conductivity (Coupel-Ledru et al., 2017). Yet, as a potentially relevant trait for water stress responses, the stomatal sensitivity to ABA in grapevines might be of importance for the varieties with high Ψ_mds_ variations upon drought progression.

Different from wild species, cultivated species are very sensitive to drought and, beyond some stress thresholds, food production is not possible. It is therefore important to gain further knowledge on the responses of plant species of agricultural importance to water shortage and their consistencies in field conditions. A valuable model for water stress studies has been the grapevine (*Vitis vinifera* L.), a species domesticated around 7,000 years ago (McGovern et al., 1996) with variability in the stomatal sensitivity to water stress (Lavoie-Lamoureux et al., 2017). In contrast to usual agricultural practices, grapevines for oenological purposes are grown under controlled water deficit as a means to increase the grape berry quality (Castellarin et al., 2007; Bucchetti et al., 2011; Pastenes et al., 2014).

It has to be underlined that controversies exist regarding the Ψ_mds_ regulation in grapevines due to *i*) the difficulty in distinguishing the isohydric to anisohydric behavior between varieties (Lovisolo et al., 2010), *ii*) the inconsistent behavior in responses to drought reported for a given variety (Schultz, 2003; Pou et al., 2012; Hochberg et al., 2013; Charrier et al., 2018), and *iii*) the fact that the isohydric or anisohydric behavior has been argued to result from plant–environment interaction rather than being an intrinsic property of the plant (Hochberg et al., 2018; Feng et al., 2019). Thus, the aim of this study was to evaluate the relationship between the degree of isohydricity with the stomatal sensitivity to drought as well as leaf traits in the grapevine varieties Syrah and Carménère experiencing a similar and non-lethal Ψ_pd_ range from *veraison*. In order to seek eventual intrinsic responses from the varieties, the experiment was carried out over two seasons, at different locations each year in central Chile, with different row orientations at each location and, for Syrah, with different rootstocks during each season. In this study, we discuss the results regarding Ψ_mds_ regulation with the hydraulic traits derived from the pressure–volume curves, the stomatal sensitivity to ABA, shoot hydraulic vulnerability, and photosynthetic responses.

## Material and Methods

### Plant Material and Setting Up

The experiments were conducted in two commercial vineyards using *Vitis vinifera* L. cv Carménère, and Syrah. During the 2017 growing season, the experiment was carried out in Viñedos Emiliana SA located at Totihue (34°20’S, 70°44’O; altitude 385 m), Cachapoal Valley, Región del Libertador Bernardo O’Higgins, Chile. Carménère own-rooted and Syrah clone 100 grafted on Kober 5 BB rootstock (*V. berlandieri* P. x *V. riparia* M.) were established in 1998 and 2000, respectively. In the 2018 growing season, the experiment was conducted in Haras de Pirque, located at Maipo Alto (34°42’S, 70°36’O; altitude 680 m), Maipo Valley, Región Metropolitana, Chile. Carménère and Syrah (clone 174) were established in 1992 and 1996, respectively. In both sites, grapevines were in a planting pattern of 1 m x 2.6 m (4,000 plants ha^-1^). The rows were east–west oriented in 2017 and north–south in 2018. Both regions correspond to a Mediterranean climate with winter rain from May to October reaching nearly 293 mm in 2016 for the Viñedos Emiliana and 326 mm in 2017 for Haras de Pirque. However, 1.2 mm of rain occurred during the 2018 summer season. The soil textural was 37% clay, 39% silt, and 24% sand in Cachapoal and 37% clay, 38% silt, and 25% sand in Haras de Pirque between 0–60 cm corresponding to a clay loam (USDA) for both locations.

Grapevines were vertically trained and pruned as double Guyot with 16 to 20 shoots per plant. Shoots were supported by 3 wires for each side of the row at 0.75 m, 1.25 m, and 2 m of height allowing a vertical shoot positional trained system. Both vineyards use drip emitters for irrigation. The drip emitters were pressure compensated, discharging 4 L h^-1^, and the drip emitters were located close to the trunk of each plant at 40 cm height. Plants were irrigated from flowering during 10 to 14 hours at night with a frequency of 7–10 days between each irrigation. For each variety, three consecutive rows were selected. In the central row, eight and 10 plants were selected for 2017 and 2018, respectively, each plant was properly marked and each one was considered as an individual. The irrigation line was removed from these three rows in the first week of January in both years, and weekly measurements of gas exchange and water potential were carried out. Simultaneous evaluations were carried out on five plants under the vineyard irrigation schedule in order to maintain well-watered conditions with values of Ψ_mds_ > -0.9 MPa (van Leeuwen et al., 2009) and/or g_s_ > 200 mmol m^-2^ s^-1^ (Medrano et al., 2002), and these results were added to the post-drought analysis on photosynthetic performance (**Figure 8**).

All measurements were made on the north side of the row during 2017 and on the east side for 2018. Full *veraison* occurred on January 27^th^ 2017 and February 2^nd^ 2018. After 60 days without irrigation, the irrigation system was resumed by adding two droppers with 4 L h^-1^ discharge for each plant, maintaining the irrigation schedule from each vineyard. The amounts of irrigation from November to March on the vineyards were 2,890 m^3^ ha^-1^ and 2,170 m^3^ ha^-1^ in the Cachapoal Valley and Maipo Valley, respectively.

### Weather Variables

All the data are in the Agromet platform (Ministerio de Agricultura and Gobierno de Chile). The weather variables data were obtained from the three meteorological stations closest to each site (no more than 15 kilometers away). Air temperature (Ta), relative humidity (RH), solar radiation (Rs), precipitation (PP), and wind speed (Ws) were obtained at 1h intervals from 4 days after irrigation was stopped. ET_0_ were computed using the Penman–Monteith model (Allen et al, 1998).

### Leaf Water Potentials (Ψ) and Gas Exchange in the Field

On the day before measurement, three consecutive sun-exposed, fully expanded mature leaves located between the 5^th^ and 12^th^ internode were selected from one shoot on each plant leaving the central leaf for gas exchange, and the upper and lower leaves were taken for assessing the pre-dawn leaf water potential (Ψ_pd_) and the midday stem water potential (Ψ_mds_). The Ψ was measured with a pressure chamber (Model 615, PMS Instrument Company, USA). The leaves were placed into the pressure chamber with the petiole protruding from the chamber lid. The chamber was pressurized using a nitrogen tank, and Ψ was recorded when the initial xylem sap was observed emerging from the cut end of the petiole using a stereo microscope (model V424B, Omax, USA). The predawn water potential (Ψ_pd_) was measured before sunrise between 5:00 h and 7:00 h. The leaves were wrapped in damp towel paper, bagged, detached with a fresh razor blade, transported in a fresh cooler box, and leaves were pressurized two minutes after detachment. The Ψ_mds_ was assessed between 11:15 h and 12:45 h. For Ψ_mds_, leaves were previously enclosed in aluminized plastic bags at least 2 h before measurement, and leaves were detached from their shoot immediately after gas exchange measurement, transported in a fresh cooler box, and finally pressurized 3 minutes after detachment.

Leaf gas exchange was measured 2–4 minutes before sampling the Ψ_mds_, by means of a portable gas analyzer (CIRAS-2, PP Systems Co. Ltd., USA) with the leaf chamber placed to imitate the natural position of leaves. Leaf gas exchange was performed, leaving the cuvette for 40–60 s until reaching a steady state. CO_2_ were adjusted to 400 µmol mol^-1^ and the light, humidity, and temperature was set to ambient.

### Physiological Traits Determination

For the determination of traits related to pressure–volume curves, stem hydraulic vulnerability curves, stomatal responses to abscisic acid, and photosynthetic performance, whole shoots were sampled early in the morning, placed inside of three black plastic bags with abundant wet paper towels, and the bags were sealed and transported to the laboratory for analysis.

#### Pressure–Volume Curve Analysis

The pressure–volume curve analysis was performed as previously described by Lawren and Pasquet-Kok (2010). Briefly, before the beginning of the drought assays in both years, post-drought analysis was carried out only in Maipo valley. Whole shoots were submerged in clean tap water for 20 minutes before taking leaves for analysis. A leaf located between the 5^th^ and 12^th^ internode was cut under water with a fresh razor blade leaving a petiole of 6–8 cm long from the lamina. Later, the petiole end was submerged in filtered (0.22 µm) and degassed KCl 10 mM and CaCl_2_ 1 mM solution and rehydrated overnight until Ψ_Leaf_ was > −0.3 MPa. Turgor loss point (Ψ_TLP_), osmotic potential at full turgor (π_0_), and capacitance (C_leaf_) were determined on one leaf from seven or eight shoots from plants before drought, and from all the plants after drought experiment, using the bench-drying technique. Leaves were slowly dried over the bench at low light (PAR < 50 µmol m^-2^ s^-1^), recording the Ψ_Leaf_, and weighed periodically until the Ψ_Leaf_ was lower than -2.5 MPa. At this point, leaves were scanned and then dried in an oven at 70 °C for 72 h to obtain leaf area and dry weight, respectively. For each leaf, the relative water content (RWC) was determined, and the changes in Ψ_Leaf_^-1^ values were plotted against 1-RWC. The Ψ_TLP_ was determined as the point of inflection between the linear and non-linear portions of the plot, the π_0_ was obtained by extrapolating the linear relationship of the post-turgor loss portion of the curve at RWC = 1 (Tyree and Hammel, 1972). Finally, the absolute capacitance per leaf area at full turgor (C_leaf_) was calculated from the slope of the linear portion from RWC and Ψ_Leaf_ before the Ψ_TLP_, normalized to saturated water content and leaf area.

#### Stem Hydraulic Vulnerability Curves

Vulnerability curves were performed using 27 and 35 shoots from different irrigated Carménère and Syrah plants, respectively, during the March of 2018. Each shoot was slowly dried over the bench under 50 µmol m^-2^ s^-1^ of light for different periods of time until reaching Ψ_stem_ values that induced embolism. The shoots were then newly bagged for 20 min to equilibrate the Ψ_leaf_ with the Ψ_stem_, and the Ψ_stem_ was recorded in two consecutive leaves up to the seventh internode. Thereafter, the shoots were submerged under 4 cm of water for 20 min to relax the tension (Hochberg et al., 2016), and immediately after, the shoot was cut, alternating at basal and apical end, with a 20 s delay between cuttings (Torres-Ruiz et al., 2015) until a shoot section longer than 9 cm located from the 9^th^ to 12^th^ internode was reached. Both ends of the sample were cut with a clean razor blade and connected to XYL’EM system (Bronkhorst, Montigny-les-Cormeilles, France) for the hydraulic measurements. After a steady flow of filtered (0.22 µm) and degassed KCl 10 mM and CaCl_2_ 1 mM solution was reached, the mean flow rate was calculated as the average of five values measured at 5 s intervals. Flow rate was measured at four different water pressure gradients ranging from 1 to 6 kPa and the hydraulic conductance of the sample (K_i_) was calculated as the slope of the flow rate versus the applied pressure verifying the linearity of the relation (Torres-Ruiz et al., 2012). Subsequently, the sample was flushed at 0.12 MPa for 5 min to remove embolism, and the maximum hydraulic conductance (K_max_) was measured. The percentage loss of conductance (PLC) was calculated as:

$$\mathrm{PLC}=100\times \left(1-\frac{K_{i}}{K_{\text{max}}}\right)$$

#### Stomatal Response to Abscisic Acid (ABA)

The stomatal responses to exogenous ABA dosages were assessed in eight leaves from the two varieties during 2018. Whole shoots from different irrigated plants were submerged in clean tap water for 20 minutes before taking the leaf for analysis. A leaf located between the 5^th^ and the 12^th^ internode was successively cut under filtered and degassed water with a fresh razor blade, leaving a petiole of 2 cm long from their lamina. The petiole was covered with parafilm, the end was refreshed with a razor blade, and it was inserted in a silicon tube of 6 cm, which was adjusted with plastic clamps. Thereafter, the silicon tubing was carefully filled with filtered (0.22 µm) and degassed KCl 10 mM and CaCl_2_ 1 mM solution by means of a syringe in order to avoid bubble formation. Then, it was submerged in the same solution in 25 mL flasks and left to rehydrate overnight. The next morning, leaves were illumined under 800 µmol photons m^-2^ s^-1^ for 1 h using a led lamp (F1A100, COMPACTA, Chile) at room temperature. Stomatal conductance (g_s_) was measured using a portable gas analyzer (CIRAS-2, PP Systems Co. Ltd., USA), leaving the cuvette for 5 min until reaching a steady state. The cuvette environment was set to 400 µmol mol^-1^ CO_2_ and 9 – 10 mbar H_2_O at room temperature. The cuvette was moved closer to the led lamp until reaching a PAR of 1,000 µmol photons m^-2^ s^-1^. After the g_s_ was recorded, the solution in the silicon tubing was discarded and it was changed for filtered and degassed 5 µM of ABA. The leaves were allowed to transpire under 800 µmol photons m^-2^ s^-1^ at room temperature for 45 min and then the g_s_ was recorded again as described before. This sequence was repeated, increasing the ABA concentration as follows: 25, 50, 100, and 150 µM. The % of g_closure_ was calculated as: 

$$\%g_{\text{closure}}=100\times \left(1-\frac{g_{\mathrm{ABA}}}{g_{0}}\right)$$

where g_0_ is the g_s_ at 0 µM of ABA for each leaf and g_ABA_ is the g_s_ at each ABA dose.

#### Drought Effects on Photosynthetic Performance

The effect of drought on photosynthetic performance was measured in detached leaves collected from the plants after irrigation was replenished during March 2018. The same procedure described in 2.4.3. was used for preparing the leaves. Leaves were illuminated at 800 µmol photons m^-2^ s^-1^ for 1 h using a led lamp (F1A100, COMPACTA, Chile) to induce stomatal opening and the activation of photosynthesis. Thereafter, the response of A to Ci was performed using a portable gas analyzer (CIRAS-2, PP Systems, USA), setting the cuvette air flux to 200 mL min^-1^ and humidity at 50%. The room temperature was controlled at 25 °C and the measurements were made at 1,000 µmol m^-2^ s^-1^ PAR using an external led lamp (F1A100, COMPACTA, Chile). The leaf was adapted to cuvette conditions as follows, 400 µmol mol^-1^ CO_2_ for 5 min, 150 µmol mol^-1^ CO_2_ for 2 min, and 30 µmol mol^-1^ CO_2_ for 15 min. Then, the gas exchange was recorded, increasing the CO2 from 50 to 400 µmol mol^-1^ in steps of 50 µmol mol^-1^, and each concentration was equilibrated for 3 min. Therefore, the CO2 was raised as follows: 500, 600, 750, 900, 1,050, 1,200, 1,400. and 1,600 µmol mol^-1^ with a 4 min interval for each concentration. The maximum rate of carboxylation (Vc_max_) and the maximum rate of electron transport at PAR = 1,000 µmol m^-2^ s^-1^ (J_max_) were estimated from the A–Ci curve data using the ‘plantecophys’ package adjusting a bilinear fitting method (Duursma, 2015).

### Data Analysis

Data analysis were performed using R software (version 3.3.3, R core team 2017). The relationships between Ψ_pd_ and Ψ_mds_ were fitted using the function *lme* in the ‘*nlme*’ package (Pinheiro and Bates, 2000) including each plant as random factor. The paired comparisons and confidence intervals between parameters of linear and non-linear regressions were obtained using the package ‘*emmeans*’ (Lenth et al., 2019). The relationships between the g_s_ and PLC responses to decreasing Ψ were fitted with a weibull function in the ‘*fitplc*’ package (Duursma, 2014). Differences in the slopes or Ψ thresholds were deemed to be significant if there was no overlap in the confidence intervals at 95%. The g_s_ response to ABA were fitted with the function *nmle* using a self-starting Michaelis-Menten model from the ´*nlme*´ package (Pinheiro and Bates, 2000). All tests fulfilled the assumptions of residual normality. Pressure volume results were compared using an ANOVA and a post hoc Tukey HSD test (P<0.05). For parameters obtained from linear and nonlinear regression, the mean and confidence interval at 95% are presented, and for weather, pressure volume, and Ψ_mds_ analysis, the mean ± SD are reported.

## Results

### Weather Conditions

**Figure 1:**
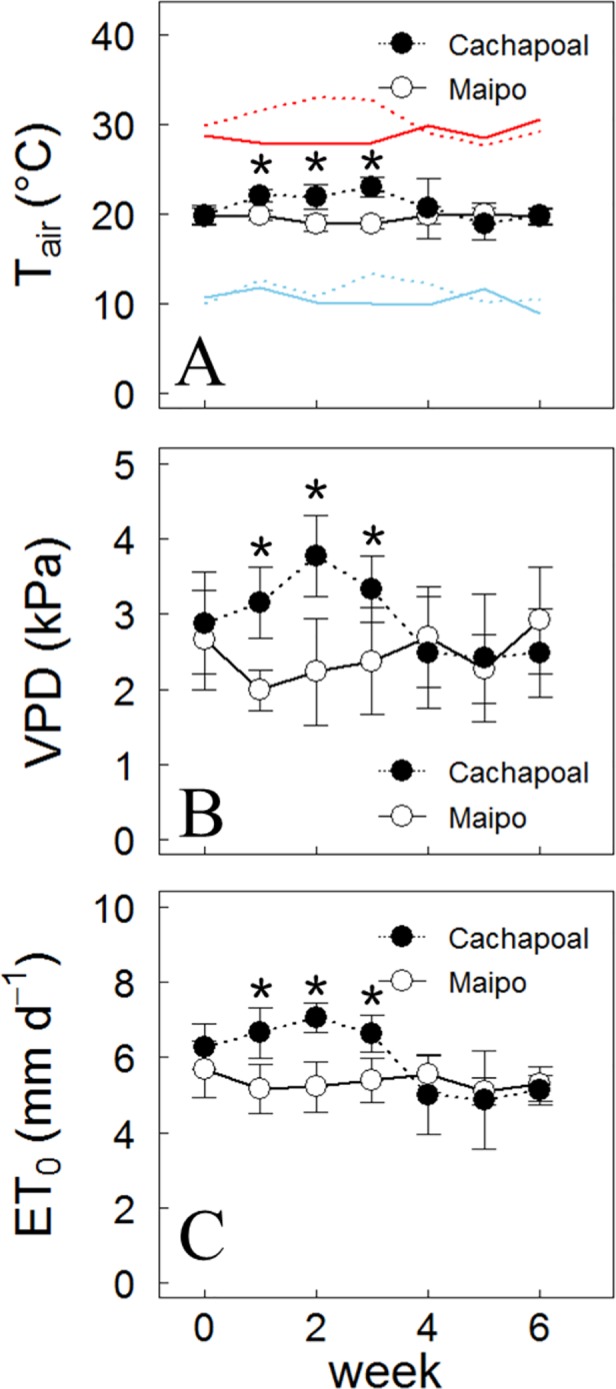
Climate variables during drought experiment. Weekly mean values of **(A)** air temperature, **(B)** vapor pressure deficit (VPD), and **(C)** reference evapotranspiration (ET_0_) through drought assays. Black symbols with dotted lines correspond to climate data from a season in the Cachapoal Valley and white symbol with continuous lines to a Maipo Valley season. Red and blue lines in A depicts the maximum and minimum temperatures. Mean weekly values ± S.D. are displayed. *Denotes significant differences between valleys (p < 0.05)

The atmospheric conditions in both locations were dry and hot and without significant rainfall during drought assays. Also, the day mean air temperature and ambient vapor pressure deficit (VPD) were 3°C and 1.21 kPa higher in the location in Cachapoal than in Maipo during their first 3 weeks of drought treatments, and then no differences were observed (**Figures 1A, B**). The minimum and maximum average temperatures were 12 °C / 31°C in the Cachapoal and 10°C / 29°C for Maipo. The higher temperature and atmospheric demand observed in Cachapoal over Maipo results in a higher reference evapotranspiration (ET_0_) for the former location than those in Maipo with a mean week values of 6.8 mm d^-1^ in Cachapoal and 5.3 mm d^-1^ in Maipo (**Figure 1C**), giving an accumulated values of ET_0_ during the assays in Cachapoal valley of 285 mm and 255 mm for Maipo valley.

### Ψ_mds_ to VPD and Ψ_pd_ Relationship in Grapevines

As shown in **Table 1**, although Carménère plants showed higher mean values of Ψ_mds_ than Syrah plants during drought experiments in both locations, the plant environment had the greatest significant effect on Ψ_mds_ (F=119) compared to the effect of varieties (F=48), with no significant interaction between variety and valley. The mean values of Ψ_mds_ for Carménère were -1.19 ± 0.18 MPa in the Cachapoal Valley and -0.91 ± 0.17 MPa in the Maipo Valley while those for Syrah were -1.46 ± 0.18 MPa and -1.07 ± 0.13 MPa in the Cachapoal Valley and the Maipo Valley, respectively (**Table 1**).

**Table 1:** Summary of midday stem water potential (Ψ_mds_) in Carménère and Syrah during drought experiments.

		Ψ_mds_		
		(MPa)		
***Variety***		mean ± SD		
Carménère		-1.02 ± 0.22		
Syrah		-1.23 ± 0.24		
***Valley***				
Cachapoal		-1.33 ± 0.23		
Maipo		-0.99 ± 0.17		
***Variety x Valley***	**Cachapoal**		**Maipo**	
Carménère	-1.19 ± 0.18		-0.91 ± 0.17	
Syrah	-1.46 ± 0.18		-1.07 ± 0.13	
***Fixed effects***	df	F		p-value
*Variety*	32	48		<.001
*Site*	32	119		<.001
*Variety x Site*	32	3.4		0.073

**Figure 2:**
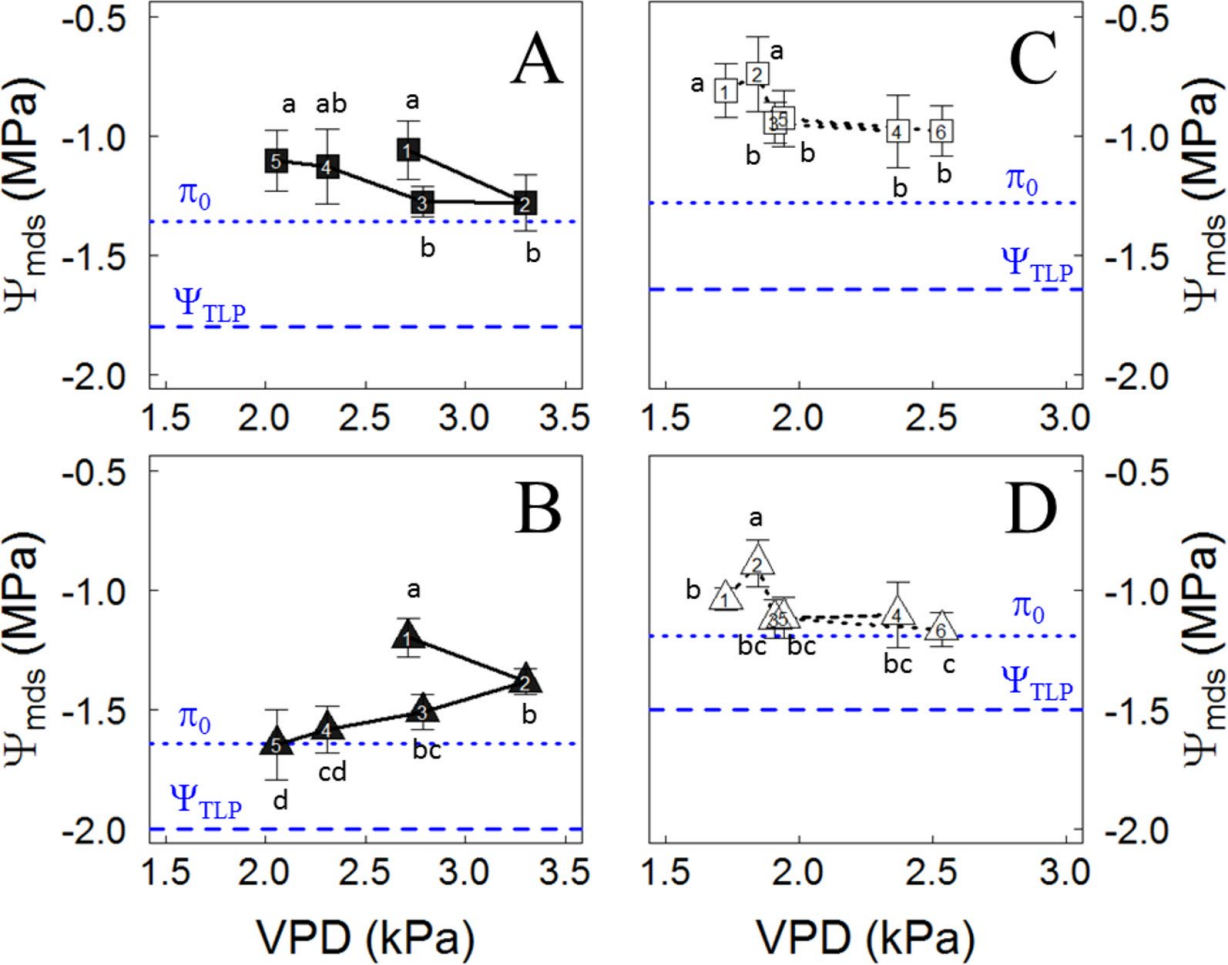
Midday water potential (Ψ_mds_) responses to vapor pressure deficit (VPD) in Carménère (squares) and Syrah (triangles) grapevines without irrigation in the Cachapoal **(A** and **B)** and Maipo **(C** and **D)**. The number inside each symbol represent the week of drought progression. The blue segmented line corresponds to the values of osmotic potential at full turgor (π_0_) and the water potential at the turgor loss point (Ψ_TLP_), shown in **Table 3**. Different letters indicate statistically significant differences between weeks of drought for the varieties in each site. Mean ± SD are presented.

As shown in **Figure 2**, after irrigation was stopped, the weekly mean of Ψ_mds_ showed different responses to VPD between varieties in the plants located in the Cachapoal Valley, showing increases in Ψ_mds_ as VPD was lowering for Carménère, as seen from the second to fifth week (**Figure 2A**), while those in Syrah showed a constant decrease in the weekly Ψ_mds_ (**Figure 2B**). On the other hand, during drought experiment in the Maipo Valley, the Ψ_mds_ showed similar trends in both varieties, showing a decrease in the Ψ_mds_ from the second to third week, and then no differences were observed in the Ψ_mds_ until the sixth week. However, the Ψ_mds_ decreased from the second to third week, from -0.74 ± 0.16 MPa to -0.94 ± 0.08 MPa in Carménère (**Figure 2C**), while in Syrah it dropped from -0.89 ± 0.09 MPa to -1.12 ± 0.08 MPa (**Figure 2D**).

**Figure 3:**
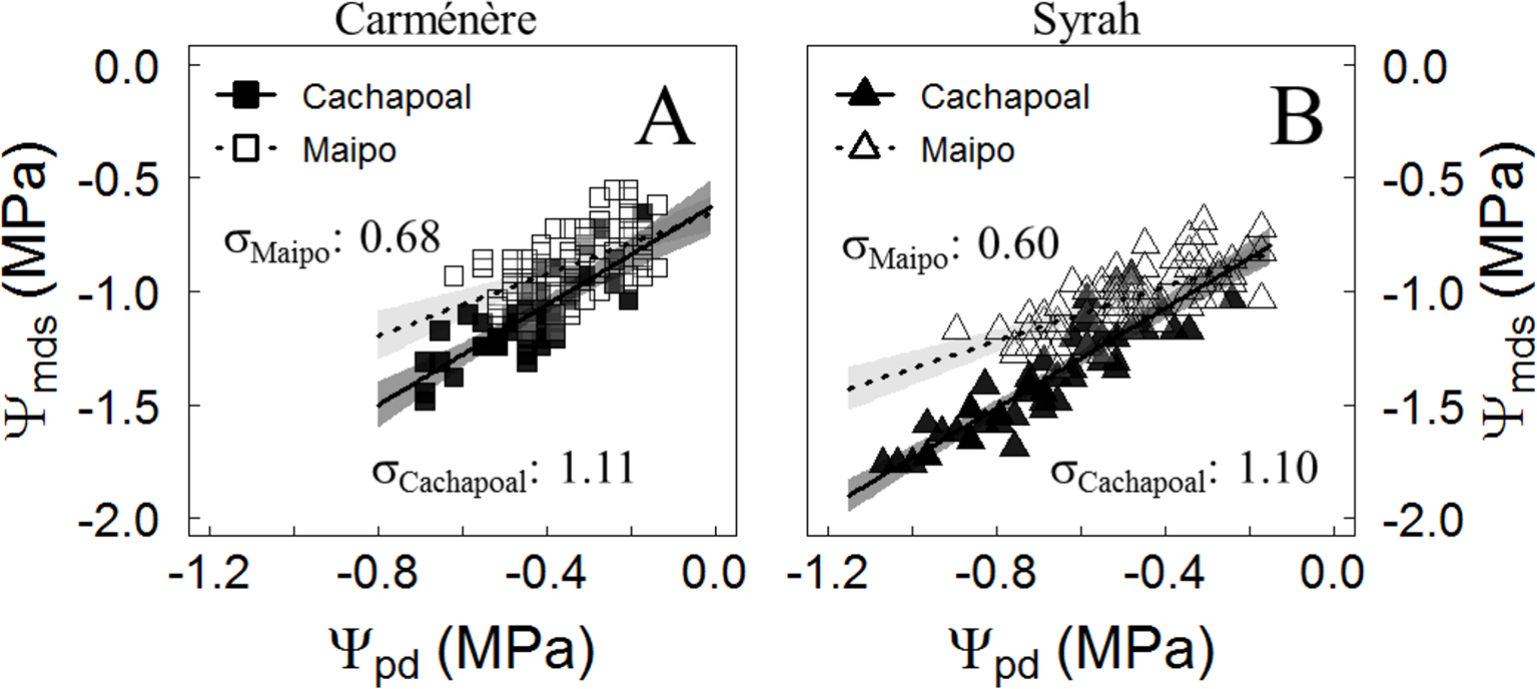
Midday water potential (Ψ_mds_) responses to predawn water potential (Ψ_pd_) in Carménère and Syrah grapevines. Trajectories from the relationship between Ψ_pd_ and Ψ_mds_ were used to estimate the degree of isohydricity in **(A)** Carménère and **(B)** Syrah plants during drought progression in the field in the Cachapoal (2017) and Maipo (2018) valleys in Chile. Squares correspond to Carménère and triangles are Syrah. Black and white symbols correspond to data from the Cachapoal Valley and Maipo Valley, respectively. The solid line shows the regression line, and the shaded area is the 95% confidence interval for the mean.

Grapevine varieties have been classified along the isohydric to anisohydric continuum, according to the strategy to manage their midday stem water potential (Ψ_mds_) upon drought. Based on their Ψ_mds_ to predawn leaf water potential (Ψ_pd_) relationship, the isohydricity degree (σ) (**Figure 3**, **Table 1**) showed a higher value in Cachapoal compared to Maipo in Carménère (p-value: 0.02) and Syrah (p-value < 0.001). Also, the Ψ_mds_ to Ψ_pd_ relationship resulted in a linear fit (**Figure 2**, **Table 1**), indicating a constant σ, irrespective of season, along the Ψ_pd_ range, which is common in viticultural practices. Carménère (**Figure 3A**) showed a σ value of 1.11 MPa MPa^-1^ (IC_95%_: 0.84, 1.37) in Cachapoal and 0.68 MPa MPa^-1^ (IC_95%_: 0.46, 0.90) in Maipo (**Table 2**). Also, the Ψ_pd_ values were reduced from -0.2 MPa to -0.7 MPa in the Cachapoal Valley and from -0.15 MPa to -0.6 MPa in the Maipo Valley (**Figure 3A**). For Syrah (**Figure 3B**), the values of σ were 1.10 MPa MPa^-1^ (IC_95%_: 0.96, 1.25) and 0.60 MPa MPa^-1^ (IC_95%_: 0.46, 0.74) in the Cachapoal and Maipo valleys (**Table 1**), respectively, with Ψ_pd_ values ranging from -0.3 MPa to -1.1 MPa in Cachapoal and -0.2 MPa to -0.9 MPa in Maipo (**Figure 3B**).

**Table 2:** Summary of statistical analysis of isohydricity degree (σ) in grapevines.

	Carménère		Syrah	
	Cachapoal Valley	Maipo Valley	Cachapoal Valley	Maipo Valley
Ψ_mds_ to Ψ_pd_				
σ	1.11	0.68	1.10	0.60
σ (IC_95%_)	(0.84, 1.37)	(0.46, 0.90)	(0.96, 1.25)	(0.46, 0.74)
p-value	0.020		<0.001	
R^2^ marginal	0.66		0.86	
R^2^ conditional	0.63		0.84	
rmse	0.11		0.08	

The slopes σ and parameters from the fit in the linear mixed model analysis (lme) are presented. The confidence interval at 95% was obtained from predicted marginal means using an emmeans package.

### Stomatal Responses to Midday Stem Water Potential and Pressure–Volume Curves in Pre-Drought Leaves

**Figure 4:**
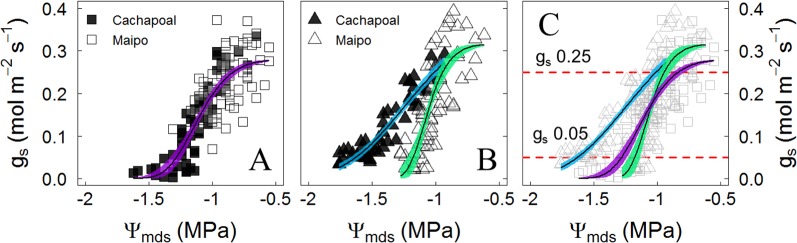
Stomatal conductance (gs) response to Ψ_mds_ in **(A)** Carménère, **(B)** Syrah, and **(C)** all plants during drought progression in the field in the Cachapoal (Cachapoal valley) and Maipo (2018) valleys in Chile. Red and Purple squares correspond to Carménère in 2017 and 2018, respectively, and light blue and green triangles are Syrah in 2017 and 2018, respectively. Solid line shows the non-linear regression, and the colored area is the 95% confidence interval for the mean. Upper and lower segmented red lines in C indicate g_s_ value at 0.25 mol m^-2^ s^-1^ and 0.05 mol m^-2^ s^-1^.

Due to the similar trajectories obtained in the changes of stomatal conductance to water potential in Carménère in both sites, the data on this variety were pooled to obtain one single regression of the stomatal response to drought (**Figure 4A**). The stomatal response to drought was significantly different between location in Syrah (**Figure 4B**). The stomatal sensitivity to drought, which corresponds to the slope of the g_s_ to Ψ_mds_ regression at 50% of the maximal g_s_, was lower in Syrah in the Cachapoal valley with a value of 121 mmol m^-2^ s^-1^ MPa ^-1^ (IC_95%_: 99, 148) compared to those in both sites in Carménère and Syrah from the Maipo Valley, with values of 184 mmol m^-2^ s^-1^ MPa ^-1^ (IC_95%_: 0.84, 1.37) and 241 mmol m^-2^ s^-1^ MPa ^-1^ (IC_95%_: 0.84, 1.37), respectively (**Table 3**). Also, as shown in **Figure 4** and **Table 2**, the Ψ_gs12_ value, which corresponds to the Ψ resulting in a 12% of the maximal g_s_, the plants of Syrah in the Cachapoal Valley were significantly more negative with a value of -1.67 MPa (IC_95%_: -1.62, -1.72) as compared to those in Syrah in the Maipo Valley and in Carménère in both sites, with Ψ_gs12_ values of -1.23 MPa (IC_95%_: -1.18, -1.23) and -1.33 MPa (IC_95%_: -1.27, -1.37). Different trajectories were observed between varieties, however, with three different regions having been observed: (1) when g_s_ > 0.25 mol m^-2^ s^-1^, g_s_ is barely regulated by Ψ_mds_ (**Figure 4C**); (2) 0.25 mol m^-2^ s^-1^ > g_s_ > 0.05 mol m^-2^ s^-1^, g_s_ is highly regulated by Ψ_mds_ (**Figure 4C**); and (3) g_s_ < 0.05 mol m^-2^ s^-1^, g_s_ values were close to Ψ_gs12_ (**Figure 4C**), corresponding, in turn, to a severe drought conditions for C3 plants(Medrano et al. 2002).

**Table 3:** Summary of hydraulic traits in grapevines of Carménère and Syrah during a season in the Cachapoal Valley and the Maipo Valley.

		*Carménère*				*Syrah*			
		Cachapoal		Cachapoal		Cachapoal		Maipo	
***Hydraulic trait***									
***Stomatal responses***									
Sx_gs50_	(mmol m^-2^ s^-1^ MPa^-1^)	198	(a)	180	(a)	121	(b)	241	(a)
Ψ_gs12_	(MPa)	-1.33	(a)			-1.67	(b)	-1.23	(a)
***Pressure-volume***									
π_0_	(MPa)	-1.36	(c)	-1.28	(b)	-1.64	(d)	-1.19	(a)
Ψ_TLP_	(MPa)	-1.80	(b)	-1.67	(b)	-2.00	(c)	-1.50	(a)
Capacitance	(mol m^-2^ MPa^-1^)	0.84	(b)	0.93	(b)	1.09	(a)	0.89	(b)
***Shoot PLC***									
Ψ_PLC12_	(MPa)			-0.74	(a)			-1.34	(b)
Ψ_PLC50_	(MPa)			-2.09	(a)			-2.62	(b)

Different letters indicate significant differences for the hydraulic traits observed between grapevines (p < 0.05).

The leaf traits obtained from pressure–volume analysis before withholding irrigation showed the highest and lowest values of both π_0_ and Ψ_TLP_ in plants of Syrah in the Cachapoal and Maipo valleys, respectively, while those in Carménère reached intermediate values in both locations (**Table 3**). The π_0_ values in Syrah were -1.64 ± 0.09 MPa and -1.19 ± 0.04 MPa for the Cachapoal and Maipo valleys, respectively, while in Carménère, π_0_ values were of -1.36 ± 0.02 MPa and -1.28 ± 0.05 MPa in Cachapoal and Maipo, respectively (**Table 1**). Also, the Ψ_TLP_ values in Syrah were -2.00 ± 0.17 MPa in Cachapoal and -1.50 ± 0.05 MPa in the Maipo Valley. As for Carménère, the Ψ_TLP_ values were -1.80 ± 0.04 MPa in Cachapoal and -1.67 ± 0.05 MPa in Maipo, but no significant differences were observed between locations for this variety (**Table 3**). The higher leaf capacitance was observed in the Syrah from the Cachapoal Valley, as compared to those in Syrah from the Maipo Valley and Carménère in both seasons. The capacitance values were of 1.09 ± 0.10 mol m^-2^ MPa^-1^ for Syrah in the Cachapoal Valley, 0.89 ± 0.09 mol m^-2^ MPa^-1^ for Syrah in the Maipo Valley, 0.84 ± 0.05 mol m^-2^ MPa^-1^ for Carménère in the Cachapoal Valley, and 0.93 ± 0.05 mol m^-2^ MPa^-1^ for Carménère in the Maipo Valley (**Table 1**).

**Figure 5:**
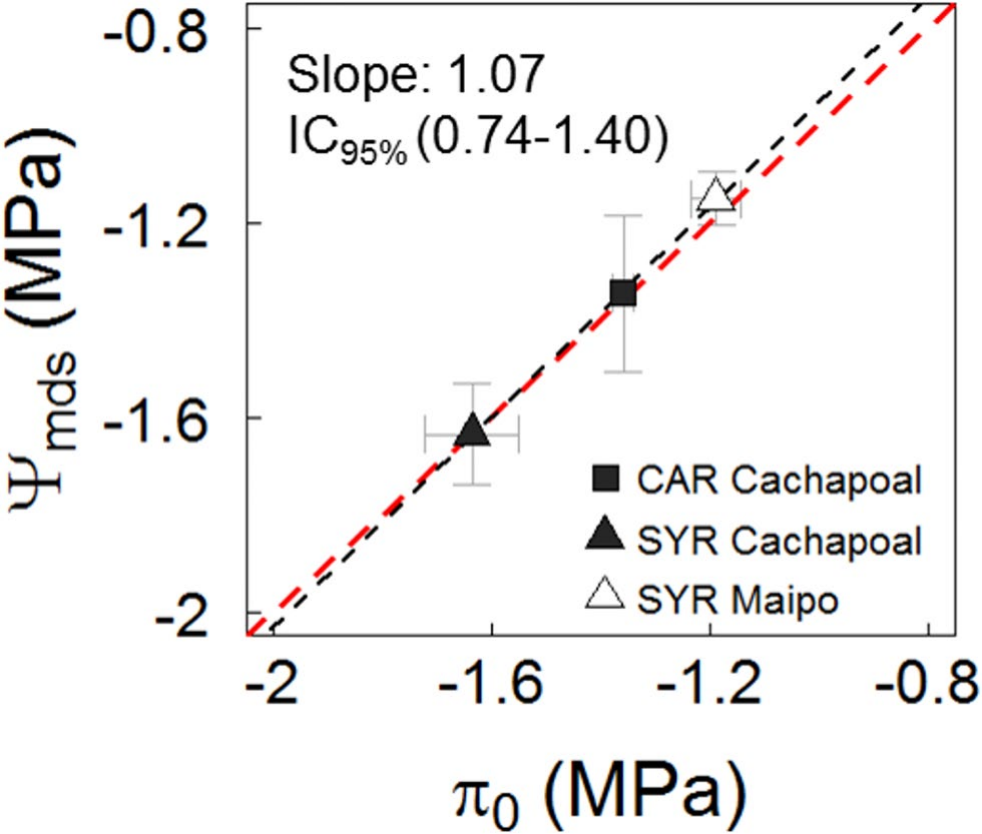
Relationship between mean midday water potential (Ψ_mds_) observed in leaves at values g_s_ < 0.05 mmol m^-2^ s^-1^ and the osmotic potential at full turgor (π_0_) in predrought leaves. Black and red line are fit and 1:1 relationship. The slope and confidence interval at 95% are presented.

**Figure 5** depicts the mean water potential values, corresponding to the g_s_ ranges lower than 0.05 mol m^-2^ s^-1^ (taken from **Figure 4**) and plotted against the related osmotic potential at full turgor (π_0_). The water potential from the plants experiencing a g_s_ < 0.05 mmol m^-2^ s^-1^ fits a nearly 1:1 ratio to the π_0_ from the leaves obtained before the drought assays were started (**Figure 5**). To some extent, however, there were similarities between Ψ_gs12_ and π_0_ (**Table 3**).

### Shoot PLC and g_s_ Sensitivity to ABA

**Figure 6:**
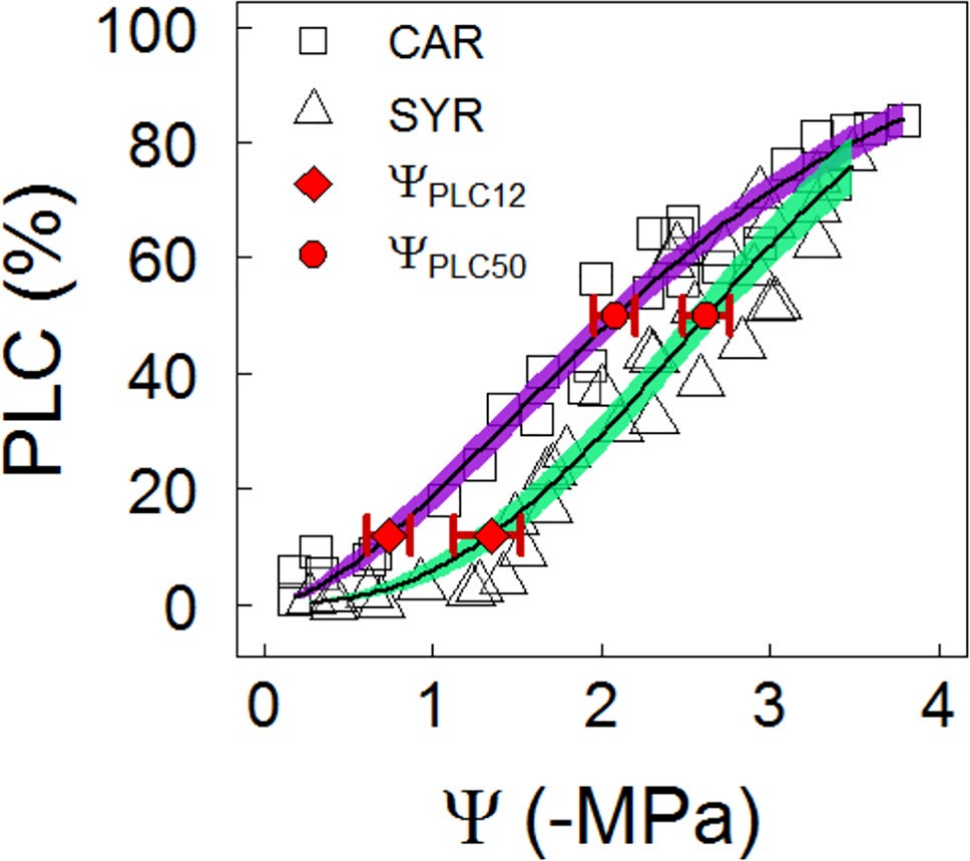
Shoots vulnerability curves of grapevines under a slow drying over a bench. Purple squares and green triangles represent the relationship between PLC and Ψ in Carménère and Syrah, respectively, from the Maipo Valley. For each curve, the predicted mean (black solid line) and the 95 % CI (colored areas) are shown. A difference in Ψ_PLC12_ (red diamond) and Ψ_PLC50_ (red circle) between varieties was deemed to be significant due to the lack of overlap between CIs (red bars).

The stem vulnerability was lower in Syrah than Carménère at the end of the season in the Maipo Valley, as determined by the bench-drying technique. Despite this, no significant differences between varieties were observed in the sensitivity to changes in water potential at the 50% loss of hydraulic conductivity (S_50_), with values of 27 ± 3 MPa^-1^ for Carménère and 33 ± 3 MPa^-1^ in Syrah. The stem vulnerability was significantly lower in Syrah compared to Carménère. Indeed, Ψ_PLC50_ values of -2.09 ± 0.07 MPa for Carménère and -2.62 ± 0.05 MPa for Syrah were observed (**Figure 6**. **Table 3**). Finally, the onset of embolism was estimated as the 12% loss of conductance (Ψ_PLC12_) with values of -0.74 ± 0.10 MPa and -1.34 ± 0.08 MPa for Carménère and Syrah, respectively (**Figure 4** and **Table 3**).

**Figure 7:**
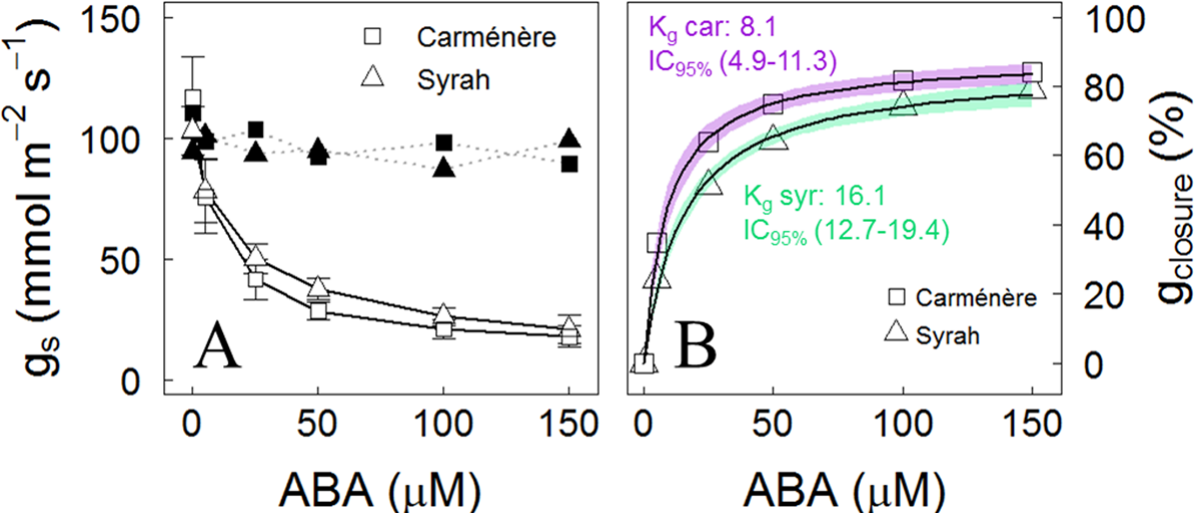
Stomatal response to increasing doses of ABA in grapevine leaves. **(A)** Average stomatal conductance when individual leaves of Carménère (squares) and Syrah (triangles) were fed with increasing doses of 0, 5, 25, 50, 100, and 150 µM of ABA for 50 min at each concentration. White symbols with straight line represent the progression response of g_s_ to increasing ABA doses and black symbols with segmented lined shows g_s_ at equal periods but in leaves fed with water as control. Mean ± SD are presented, n: 7. **(B)** Percentage of stomatal conductance reduction (g_closure_) from the initial value from each leaf. The g_closure_ response to ABA was fitted using a Michaelis–Menten curve. For each curve, the predicted mean (black solid line) and the 95 % CI (segmented lines) are shown. The ABA concentration at which g_s_ is reduced to its half (K_g_) for each variety are presented.

Syrah leaves fed through their transpiration stream with ABA showed a similar stomatal response to those of Carménère (**Figure 7**). Even though both varieties reached g_s_ values of near 20 mmol m^-2^ s^-1^ at 150 µM of ABA and the percentage of the maximum stomatal closure (g_max closure_) was no different between varieties, with a mean value of 85%, the ABA concentration,l in which g_max closure_ is reduced to their half (K_g_), was lower in Carménère than Syrah, with values of 8.1 µM (IC_95%_: 4.9, 11.3) and 16.1 µM (IC_95%_: 12.7, 19.4), respectively.

### Post-Drought Effects on the Photosynthetic Performance

**Figure 8:**
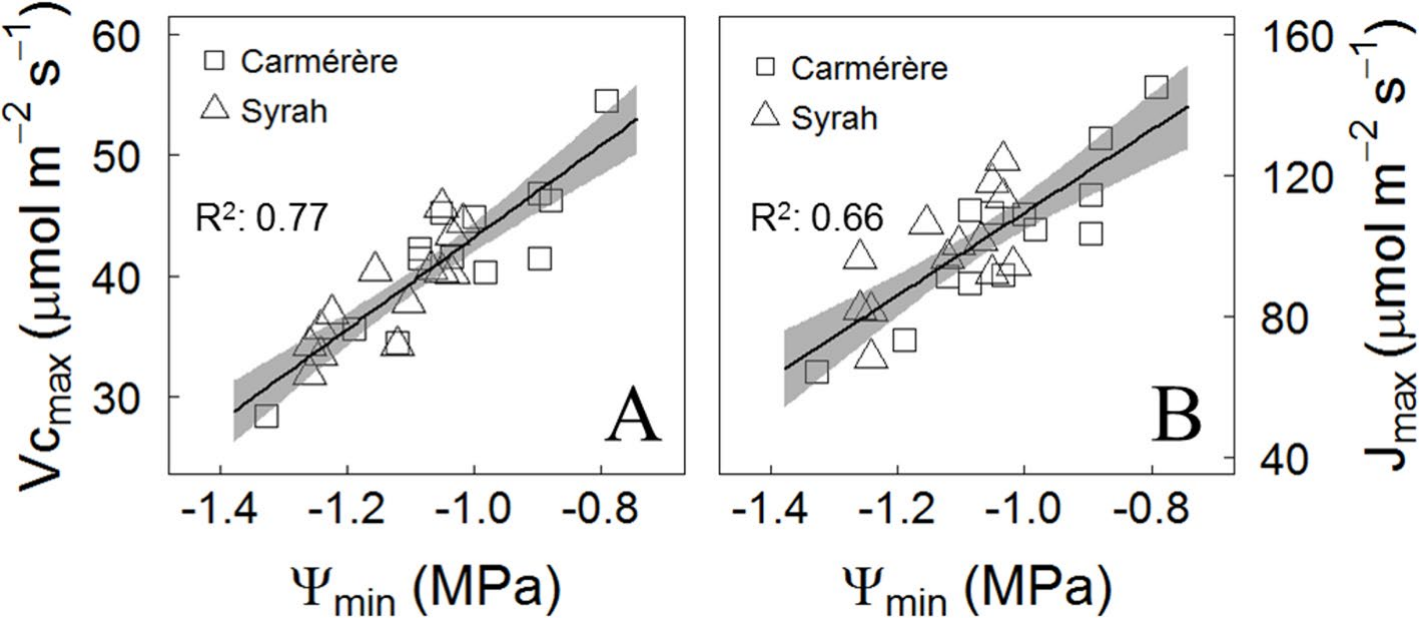
Post-drought effects on the photosynthetic performance. **(A)** Response of Maximum rate of carboxylation and **(B)** Maximum rate of electron transport at photosynthetically active radiation of 1,000 μmol m-2 s-1 to the mean of two lowest values of Ψ_mds_ (Ψ_min_) registered for each plant over the season. The determination coefficient (R2) is presented.

At the end of the season in control and droughted plants located in the Maipo Valley, the relationship between the minimum Ψ_mds_ (Ψ_min_) values recorded during the season and the photosynthetic performance of the varieties, derived from the A/Ci curves after irrigation was resumed during March 2018, were analyzed for each plant. As for the maximum rate of carboxylation (Vc_max_) and the maximum electron transport rate (J_max_), both were reduced upon progressively more negative Ψ_min_ values, but with no differences between varieties (**Figures 8A, B**). The Vc_max_ and J_max_ to Ψ_min_ ratios were 38 µmol m^-2^ s^-1^ MPa^-1^ and 117 µmol m^-2^ s^-1^ MPa^-1^, respectively (**Figures 8A, B**).

## Discussion

Considering the importance of the plant water potential on growth and metabolism, it is relevant to know how plant species respond to water scarcity, particularly in grapevines; they are a species often cultivated under controlled water deficit conditions with contrasting responses between varieties in their water status under stress (Bota et al., 2016; Lavoie-Lamoureux et al., 2017). Such responses have led to the classification of grapevine varieties in the iso/anisohydric continuum according to their capacity to regulate Ψ_mds_. But, Ψ_mds_ results from both the soil water status and the transpiration rate linked to the evaporative demand (Tardieu and Simonneau, 1998). As shown in **Figure 1**, the weather was hotter and drier, resulting in a higher water demand from the air in the Cachapoal Valley than that in the Maipo Valley. Concomitantly, significantly lower Ψ_mds_ values during the drought progression in both varieties were observed in the Cachapoal Valley compared to the Maipo Valley (**Table 1**).

A complication for the classification of species and varieties in the iso/anisohydric continuum lies in the fact that, as previously shown, plants may shift their strategy for regulating their water status, from anisohydric to isohydric (Meinzer et al., 2016; Charrier et al., 2018), as assessed by the Ψ_pd_ to Ψ_mds_ ratio in a season. These has been observed in grapevines under a drought progression together with an increasing VPD condition (Collins et al., 2010). If that would be the case, rather than a constant σ, a reduction of its value should be observed along the drought progression, as reported before in Sangiovese (Poni et al., 2007), Grenache and Syrah (Charrier et al., 2018), as well as in some *Quercus* species (Fu and Meinzer, 2018; Feng et al., 2019). We have assessed the Ψ_pd_ to Ψ_mds_ ratio in different study sites, row orientations, and varieties in all cases with high VPD conditions and around *veraison*, the time when deficit irrigation is practiced in Mediterranean climates because of its positive impact on berry quality (Castellarin et al., 2007), and in a range of water potential values common in viticulture. In all cases, σ resulted in being be strictly linear, suggesting that no changes in the extent of isohydricity occurs in grapevines in field conditions under a progressive water deficit (**Figure 3**, **Table 2**).

As for the environmental effect on plant–water relations, it is well known that the plant water status and stomatal behavior are both controlled by atmospheric and soil moisture conditions (Dixon and Joly, 1895; Jarvis, 1976; Sperry et al., 2002; Guo and Ogle, 2019). Ψ_mds_ is mainly affected by VPD, rather than just the Ψ_pd_ under low to moderate water deficit (Fu and Meinzer, 2018). Also, since VPD and temperature serve as a proxy for evaporative demand, both are important predictors for Ψ_mds_ in grapevines (Williams and Baeza, 2007; Brillante et al., 2016). However, it has to be underlined that a very conserved stomatal response to Ψ_mds_ was observed in Carménère (**Figure 4A**), even though the environment and row orientation were different on each location. In this sense, the degree of isohydricity (σ) by itself in Carménère (**Figure 3A**) does not necessarily result from the stomatal behavior under drought in field conditions (**Figure 4A**). On the contrary, a coordination between σ and the g_s_ response to Ψ_mds_ was noticeable for Syrah when comparing both study sites. Indeed, Syrah plants from the Maipo Valley had a lower σ (**Figure 3B**), a higher stomatal sensitivity to Ψ_mds_ (Sx_gs50_), as well as a higher Ψ_mds_ threshold for stomatal closure (Ψ_gs12_) compared to plants from the Cachapoal Valley (**Figure 4B**, **Table 3**). These contrasting results are likely to be due to a different Ψ_mds_ sensitivity to VPD between varieties.

For grapevines in the Cachapoal Valley, the σ value was similar between varieties (**Table 2**, see mean values and confidence intervals), but Carménère resulted in a higher stomatal response to Ψ_mds_ than Syrah (**Figure 4**, **Table 3**). In this study site, a higher VPD occurred along the first three weeks of the drought progression, later decreasing until the fifth week (**Figure 1**). From these, it is clear that the VPD-driven reduction in Ψ_mds_ was more pronounced in Carménère (**Figure 2A**) compared to Syrah (**Figure 2B**) as, in the latter, Ψ_mds_ was constantly reduced over time regardless of VPD (**Figure 2B**). Our results suggested that, on one hand, changes in Ψ_mds_ are mainly driven by the soil water content in Syrah and, on the other, that the differences in σ between both study sites in Syrah results from a differential sensitivity of g_s_ to Ψ_mds_ (**Figure 4B**). On the contrary, however, Ψ_mds_ (**Table 1**) as well as σ (**Figure 3A**, **Table 2**) are both rather influenced by VPD in Carménère.

If there was a differential stomatal sensitivity in Ψ_mds_, as observed in Syrah between both study sites, differences in some leaf traits, such as those derived from the pressure–volume curves, should therefore also be observed. The leaf traits obtained from pressure–volume curves have been related to drought tolerance (Maréchaux et al. 2015; Zhu et al. 2018). For instance, a high correlation has been observed between the Ψ threshold for g_s_ reductions (i.e. Ψ_gs50_, Ψ_gsclose_) and Ψ_TLP_ (Brodribb et al. 2003; Bartlett et al. 2016; Farrell et al. 2017). In Carménère vines, a similar Ψ_TLP_ was observed in both locations while π_0_ was merely 0.08 MPa higher in the plants from Cachapoal compared to those from the Maipo Valley (**Table 3**). However, in Syrah, the differences in Ψ_TLP_ and π_0_ were close to 0.5 MPa between both locations (**Table 3**), being a much more contrasting magnitude in the context of grapevines. Besides the environment, water relation responses are influenced by rootstock-scion interaction (Vandeleur et al., 2009; Gambetta et al., 2012; Marguerit et al., 2012; Tramontini et al., 2013; Gullo et al., 2018) and clonal variability (Coupel-Ledru et al., 2014; Tortosa et al., 2016) among others. Since Carménère plants were own rooted in both locations and this variety was introduced in Chile before the phylloxera crisis in Europe, showing a very low genetic diversity compared to other varieties (Moncada and Hinrichsen, 2007), the g_s_ response to Ψ_mds_ were similar.

However, there is evidence that rootstocks influence the water relations of plants under periods of drought (Soar et al., 2006a; Tramontini et al., 2013; Lavoie-Lamoureux et al., 2017) because of their effect on the capacity to extract water from drying soils, which alters the rate of fine root maturation and suberization (Barrios-Masias et al., 2015), and/or due to higher aquaporin gene expression (Gambetta et al., 2012), resulting in a greater whole-root system hydraulic conductance for vigor-promoting rootstocks. Indeed, the plants of Syrah in the Cachapoal Valley were grafted on Kober 5bb, known to promote vigor, even though a low resistance to drought has been reported (Carbonneau, 1985; Keller, 2010; Zhang et al., 2016). Similarly, the genetic diversity in Syrah may have also influenced the marked variation in σ and the g_s_ responses to Ψ_mds_ between locations as clones were different. Altogether, it is not possible to expect a putative drought response for each grapevine variety since the root structure and physiology, among other traits and environmental cues, may significantly affect both the stomatal sensitive to Ψ_mds_ and the Ψ_mds_ to Ψ_pd_ ratio. It is likely that the inconsistencies in the reported classification of grapevine varieties, especially in these case for Syrah, in the iso/anisohydric continuum is the result of such combinations (Schultz, 2003; Pou et al., 2012; Hochberg et al., 2013; Charrier et al., 2018).

In woody species, most of stomatal downregulation can be explained by turgor during dehydration under mild water stress conditions (Rodriguez-Dominguez et al. 2016), supporting the hypothesis that hydraulic signals close the stomata while ABA is important for sustaining a low gs under lower water potentials (Tombesi et al. 2015; Degu et al. 2019) or higher VPD (Speirs et al. 2013; McAdam and Brodribb, 2015), without a clear role of the effect of rootstocks in grapevines (Soar et al. 2006b; Peccoux et al. 2018). According to our results, the ABA fed through the transpiration stream in leaves reduced the g_s_ down to 80% in both varieties, but the concentration of the phytohormone needed to reduce g_s_ by half its maximum is double in Syrah compared to Carménère (**Figure 7**), suggesting a higher g_s_ sensitivity to ABA in the latter. It has been reported that ABA biosynthesis, distribution, transport and the ability to induce stomatal closure are more pronounced in leaves than roots under conditions of water stress (Hu et al. 2016; Zhang et al. 2018). Besides, ABA increases in their active form in response to transient increases in the VPD (McAdam and Brodribb 2015) and over the course of the day (Lovisolo et al. 2008; Tombesi et al. 2015; Brunetti et al. 2019). In this sense, a greater stomatal sensitivity to ABA could be associated to less water loss during the day, avoiding excessive water release from internal storage compartments and reducing the probability of reaching Ψ thresholds that induce irreversible hydraulic failure, until the environment turns more favorable and/or a deeper rooting has been reached (Creelman et al. 1990; McAdam et al. 2016; Blackman et al. 2016). Therefore, the similar g_s_ sensitivity to Ψ_mds_, as observed in 2018 in both varieties (**Figure 4**, **Table 3**), but with differences in their stomatal response to ABA, might reflect differences in their strategy to respond to a drought progression. Carménère is a variety more prone to cavitation at shoot level than Syrah (**Figure 6**), and the higher sensitivity to ABA might imply a more sustained g_s_ reduction, long enough as to allow for new root growth in case water is available from deepest soil layers after drought events. Besides, Carménère, a variety harvested much later than other red colored counterparts, is less prone to basal leaf shedding triggered by drought, contrary to what is commonly observed in Chilean commercial Syrah vineyards. However, further experiments are needed for disentangling the responses of ABA to VPD and their effects on drought resistance in plants with similar thresholds of g_s_ responses to Ψ_mds_.

Irrespective of the degree of isohydricity of the varieties, to some extent there were similarities between Ψ_gs12_ and π_0_ in plants before we withheld the irrigation over the course of both seasons (**Table 3**). This is likely due to the fact that when Ψ_mds_ reaches a value similar to π_0_, the leaf turgor Ψ (Ψ_p_) is ∼20% of its maximum (**Supplementary Figure 1**). Besides, in C3 plants, including grapevines, g_s_ has been proposed as a reference for drought intensity (Medrano et al. 2002). From **Figure 4C**, it is possible to distinguish the g_s_ response regions proposed by (Medrano et al. 2002): (1) g_s_>250 mmol m^-2^ s^-1^, where stomatal limitations to photosynthesis are defined as dominant and, from our results, g_s_ is barely regulated by Ψ_mds_ (**Figure 4C**); (2) roughly 250 mmol m^-2^ s^-1^ > g_s_ > 50 mmol m^-2^ s^-1^, where stomatal and non- stomatal limitations are thought to affect photosynthesis and, in both varieties, g_s_ is regulated by Ψ_stem_ (**Figure 4C**); and (3) g_s_ < 50 mmol m^-2^ s^-1^, with non-stomatal limitations being the dominant limitation to photosynthesis and where g_s_ reaches values close to Ψ_gs12_ (**Figure 4C**), which is interpreted as a severe drought condition for the plant. When assessing the relationship between Ψ_mds_ and π_0_ in those plants experiencing g_s_ < 50 mmol m^-2^ s^-1^, as compared to the same group of plants before the drought progression, the former fit in a nearly 1:1 ratio (**Figure 5**). These results suggest that there is no osmotic adjustment, at least during the drought period, and, therefore, π_0_ might be used as a convenient tool to define the Ψ_mds_ threshold values posing a risk in productive systems under controlled water deficit irrigation, as in grapevines for oenological purposes.

The major negative impact of stomatal closure in water-stressed plants is the detrimental effect it has on the CO_2_ concentration at the carboxylation sites. As the extent of the water deficit increases and is sustained in time, non-stomatal limitations to photosynthesis have been described. From our results, and regardless of the variety and their corresponding σ value observed in 2018 (**Figure 3**, **Table 2**), the more negative the Ψ_min_ experienced by leaves, the lower the Vc_max_ and the J_max_ after the recovery from water stress (**Figure 8**), although both varieties showed similar stomatal response to drought during 2018 (**Figure 2**). Besides the impact on mesophyll conductance (Grassi and Magnani 2005; Keenan et al. 2010), other effects have been described in water-stressed leaves, such as reductions in the content of photosynthesis-related proteins (Delatorre et al. 2008; Michaletti et al. 2018), which might explain the non-reversible reduction in the carboxylation capacity in both varieties in the present study. Also, water stress is known to reduce electron transport in the photosynthetic apparatus through damage to the Mn-cluster at the PSII electron donor side, as well as both photosystem reaction centers (Toivonen and Vidaver 1988; He et al. 1995). From our results, the non-extreme water stress conditions, normal in grapevine production for wine making, might have a non-reversible impact on plants photosynthesis.

## Conclusions

From our results, we conclude that the degree of isohydricity is not an intrinsic trait in the Syrah and Carmenre grapevine varieties since σ seems to be strongly affected by the evaporative demand from air. Also, σ is not necessarily associated with stomatal sensitivity to drought since, in Carmenere, we found differences in σ between locations but without changes in the g_s_ to Ψ_mds_ relationship. On the other hand, besides the environment, the g_s12_ and σ seems to be influenced by genetic variability or rootstock in Syrah. From our results, a multi-season common garden experiment at the same locations is needed to evaluate the grapevine responses to drought in order to better understand the implications of rootstocks and clone variability.

Independent from the degree of isohydricity and given the similarity between the π_0_ and Ψ_gs12_ in leaves previous to drought, it seems that π_0_ could be a convenient tool for assessing the Ψ_mds_ threshold values posing a risk to the plants, which is helpful for irrigation decision making in grapevines under a controlled water deficit. However, further knowledge on the capacity of the varieties to recover after reaching those Ψ_mds_ thresholds is needed.

Finally, we found that the photosynthetic capacity was linearly and irreversibly affected by the extent of drought experienced at the stem level in both varieties.

## Data Availability Statement

The datasets generated for this study are available on request to the corresponding author.

## Author Contributions

LV-G and CP conceived and planned the study. LV-G carried out the experiment, analyzed the data and wrote the first draft. LV-G and MM managed the vineyard, helped to collect and manage data. NF and CP supervised the research. CP contributed to the writing and editing of the manuscript.

## Funding

The authors thank the funding from CONICYT (FONDECYT Project N° 1140880). We also thank the funding from the CONICYT + PAI, Concurso nacional tesis de doctorado en el sector productivo, convocatoria 2016 + Folio T7816120001 and the VID short visit funding support from the Universidad de Chile. Also, we are grateful to Programa de Doctorado en Ciencias Silvoagropecuarias y Veterinarias de la Universidad de Chile for partial funding.

## Conflict of Interest

The authors declare that the research was conducted in the absence of any commercial or financial relationships that could be construed as a potential conflict of interest.
