# Supplementary material for: Controversies in Midday Water Potential Regulation and Stomatal Behavior Might Result From the Environment, Genotype, and/or Rootstock: Evidence From Carménère and Syrah Grapevine Varieties
Source: Front Plant Sci. 2019 Dec 2;10:1522. doi: 10.3389/fpls.2019.01522 (PMC6900739; doi:10.3389/fpls.2019.01522)
Supplement: Supplementary file 1 [file DataSheet_1.docx]

**Supplementary Figure 1.**


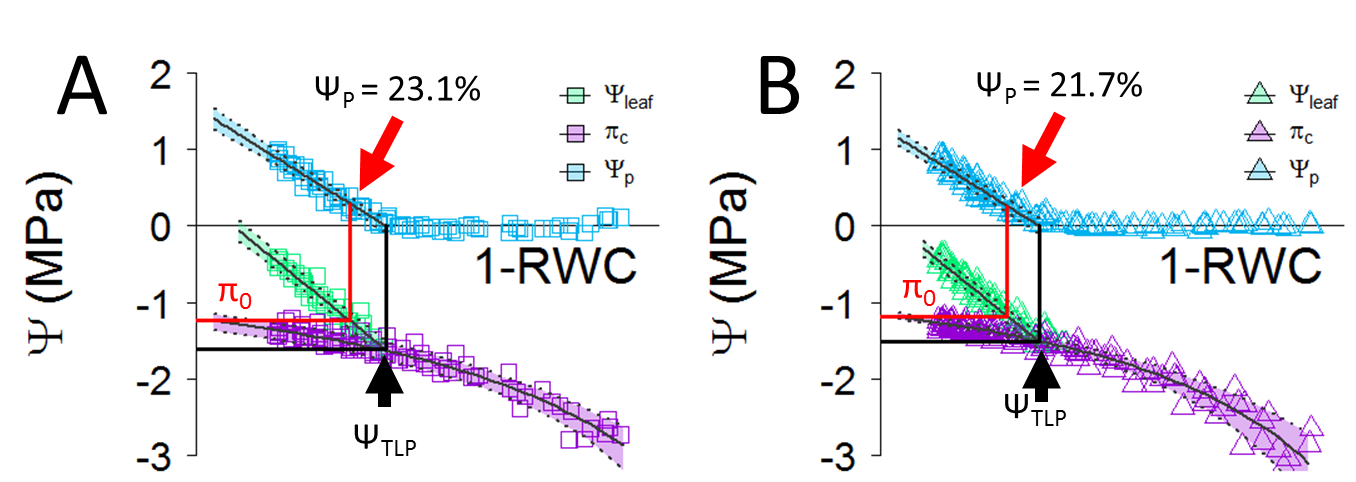


**Supplementary Figure 1.** Graphical representation of pressure-volume curves. The relationship Ψ and 1 – relative water content (RWC) from Carménère (A, squares) and Syrah (B, triangles) leaves before the irrigation withholding during the 2018 season. The leaf water potential (Ψ_leaf_) is the sum of the pressure potential (Ψ_p_) and solute potential (π_c_). Green, purple and light blue points are the Ψ_leaf_, osmotic potential (π_0_) and pressure potential (Ψ_p_), respectively. Black arrow indicates the Ψ_leaf_ at turgor loss point (Ψ_TLP_) and red arrows shows the percentage of the Ψ_p_ at Ψ_leaf_ value equal to π_0_.
